# Cohort study of consistency between the compliance with guidelines for chemotherapy-induced nausea and vomiting and patient outcome

**DOI:** 10.1186/s40360-015-0005-1

**Published:** 2015-03-28

**Authors:** Masahiro Inoue, Manabu Shoji, Naomi Shindo, Kazunori Otsuka, Masatomo Miura, Hiroyuki Shibata

**Affiliations:** Department of Clinical Oncology, Graduate School of Medicine, Akita University, Akita, Japan; Division of Chemotherapy for Outpatient, Akita University, Akita, Japan; Department of Pharmacy, Akita University, Akita, Japan

**Keywords:** Chemotherapy-induced nausea and vomiting, Guidelines, Emetogenicity

## Abstract

**Background:**

Chemotherapy-induced nausea and vomiting is one of the most influential factors that affect patient quality of life; thus, preventing this adverse event could lead to better patient outcome. Standard preventive guidelines for antiemetic treatment have already been established based on the emetogenicity of chemotherapeutic agents. It is important that compliance with in-house guidelines and their effect on patient outcome is monitored.

**Methods:**

In 3 years since the Akita university hospital antiemetic guidelines were outlined, we assessed the incidence of chemotherapy-induced nausea and vomiting using the antiemesis tool of the Multinational Association of Supportive Care in Cancer. Compliance of the guidelines was extracted from the hospital clinical record, and the chemotherapy-induced nausea and vomiting was examined by the patient reported outcome.

**Results:**

Seventy-three patients answered the questionnaire. The overall compliance rate with the guidelines for early nausea and vomiting was 98.6% and with the delayed nausea and vomiting was 87.7%. The complete response rate for the early and delayed chemotherapy-induced nausea and vomiting was 77.8% and 73.8%, respectively. The overall relative risk of early nausea and vomiting was 0.22 (P < 0.05), whereas the relative risk for delayed nausea and vomiting was 2.09 (P < 0.05). Breakthrough vomiting was observed in 3 cases in the low-risk group only. These data suggest that delayed nausea and vomiting is difficult to prevent, particularly in the low-risk group. Further, it seems that the individual sensitivity for emetogenicity might differ among patients.

**Conclusions:**

In addition to standard prevention guidelines based on emetogenicity, individual care based on patient reports should be considered for the complete prevention of chemotherapy-induced nausea and vomiting.

**Electronic supplementary material:**

The online version of this article (doi:10.1186/s40360-015-0005-1) contains supplementary material, which is available to authorized users.

## Background

One of the most devastating effects on the quality of life (QOL) of cancer patients is chemotherapy-induced nausea and vomiting (CINV). CINV is believed to affect 70%–80% of patients that receive cancer chemotherapy [1]. In addition, even effective chemotherapy may be stopped because of severe CINV, and the clinical losses may be significant in these cases [2].

The underlying mechanisms that cause CINV have been determined gradually, and many neurotransmitters such as 5-hydroxytryptamine (5-HT_3_, serotonin) and substance P are involved in CINV [3]. 5-HT3 receptor antagonists (5-HT3RA) such as granisetron, ondansetron, and palonosetron have been approved for the treatment of CINV [4]. In addition, aprepitant was approved as a substance P blocker and a neurokinin 1 (NK1) receptor antagonist (NK1RA) for the prevention of acute and delayed CINV [4]. These agents have greatly improved patient outcome regarding CINV. For example, it was reported that a complete response of CINV occurred in 53.6%–53.7% of patients that received moderately emetogenic chemotherapy using palonosetron plus dexamethasone (DEX) [5]. It was also reported that, among patients receiving cisplatin-based chemotherapy, the advantage achieved by the use of aprepitant was 20 percentage points [6].

Since the Multinational Association of Supportive Care in Cancer (MASCC) released their antiemetic guidelines in 1998, the American Society of Clinical Oncology (ASCO) and the National Comprehensive Cancer Network (NCCN) also released guidelines for the prevention and treatment of CINV [7]. Antiemetic strategies primarily using 5-HT3RA, NK1RA, and DEX have been established and recommended based on four emetogenicities (high, moderate, low, and minimal risk) for each chemotherapeutic agent [7]. In individual institutions, in-house guidelines for the prevention and treatment of CINV according to the MASCC, ASCO, and NCCN guidelines have been created and implemented, including in our hospital. Added to these guidelines, it is very important to monitor both patient outcome and their compliance with the guidelines [8]. However, because awareness of the occurrence of CINV depends only on patient declarations, it is difficult to know the severity of the CINV without patient-reported outcomes. In the current study, we assessed the consistency between the CINV compliance guidelines set in 2010 in Akita University Hospital and the outcome of patients visiting the Division of Chemotherapy for Outpatients. In this observational study, we revealed that the individual monitoring of CINV, even in low-risk emetogenicity patients, is very important to improve patient QOL during chemotherapy.

## Methods

**Figure 1:**
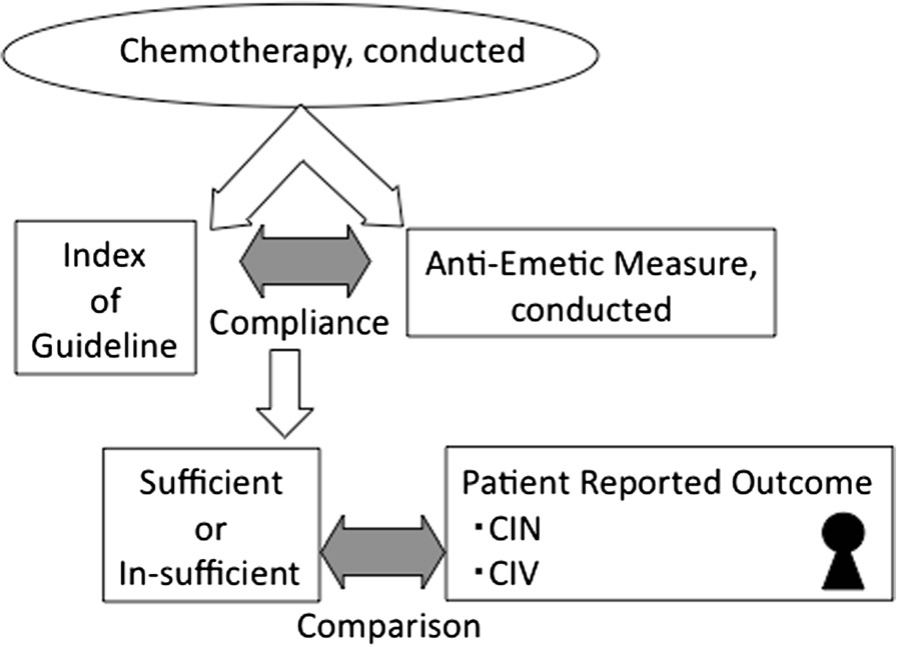
**Schematic presentation of this study.** Comparison between compliance of anti-emetic guideline and patient reported outcome.

Patients who visited the Division of Chemotherapy for Outpatients, Akita University Hospital, between November 2013 and March 2014 were asked about their early and delayed CINV using the Japanese version of the MASCC Antiemesis Tool (MAT) [9], which was administered at a usual medical examination using a questionnaire. The grade of nausea was rated from 1 to 10 (1, minimum; 10, maximum), and the number of times of vomiting was recorded by the patients themselves. Questions were asked at any time during treatment, but only once for each patient. Conducted chemotherapeutic and anti-emetic agents were extracted from electronic clinical record of hospital, and the compliance of guideline was examined. Further, comparison was made between the patient reported outcome and the compliance of guideline (Figure 1). Stat Mate III (ATMS, Tokyo, Japan) was used to calculate the relative risk. The level of statistical significance was set as *P* < 0.05. This retrospective study was approved by the Ethics Committee of the School of Medicine of Akita University. Written informed consent for participation in the study was obtained from participants.

## Results and discussion

### Patients

The patients included 36 males and 37 females aged 28–79 years, with a median age of 62 years (Table 1). The patients were diagnosed with the malignancies listed in Table 1. Colorectal cancer was most frequent (16/73), followed by breast (13/73), lung (9/73), and gastric cancers (9/73). All participants were diagnosed as suitable for chemotherapy as outpatients, without any clinical signs of brain metastasis or intestinal obstructions. The performance status of all participants was below 2, according to the Eastern Cooperative Oncology Group. The detailed clinical information such as clinical stages, previous treatment is listed in Additional file 1: Table S1. Chemotherapy naïve, and the other patients experienced ≥2 cycles of chemotherapy.

**Table 1:** **Patients’ characteristics**

**Number of participant**	73
**Age (median)**	28-79 (62)
**Male: Female**	36: 37
**Chemotherapy naïve patients**	3
**Primary leasion**	
**Esophageal cancer**	2
**Gastric cancer**	9
**Colorectal cancer**	16
**Bile duct carcinoma**	1
**Pancreatic cancer**	7
**Breast cancer**	13
**Lung cancer**	9
**Malignant lymphoma**	3
**Acute lymphoblastic leukemia**	1
**Malignant myeloma**	2
**Prostate cancer**	5
**Renal cell carcinoma**	1
**Cancer of unknown primary**	4

### Regimens

The regimens used to treat the patients and their emetic risks are listed in Table 2. The most frequent regimen was docetaxel alone (9/73), followed by gemcitabine (8/73). The regimens were divided into four emetic risk groups: high, moderate, low, and minimal risks. The number of patients receiving high-, moderate-, low-, and minimal-risk regimens was 11, 17, 38, and 7, respectively.

**Table 2:** **Treatment regimens and emetogenisity**

**Emetogenisity**	**Index of anti-emetic guideline**	**Regimen**	**Number**
**Minimal (n = 7)**	Early; (−), Delayed; (−)	Tmab + VNR	2
		Tmab	2
		VNR	1
		MTX + VCR + PSL	1
		temsirolimus	1
**Low (n = 38)**	Early; DEX, Delayed; (−)	GEM	8
		S-1 + GEM	1
		nab-PTX + GEM	1
		nab-PTX	3
		Tmab + PTX	2
		PTX	1
		DTX + S-1	2
		DTX + EMP	1
		DTX + PSL	4
		DTX	9
		eribulin	1
		Bmab + sLV5FU2	1
		sLV5FU2	3
		VTD	1
**Moderate (n = 17)**	Early; NK1RA + 5-HT_3_RA + DEX, or 5-HT_3_RA + DEX, Delayed; NK1RA + 5-HT_3_RA + DEX, or 5-HT_3_RA + DEX	Bmab + CBDCA + PTX	3
		Pmab + FOLFOX6	2
		Bmab + CapeOX	1
		CapeOX	3
		SOX	1
		Bmab + FOLFIRI	1
		Bmab + IRIS	1
		IRIS	2
		CPT-11	2
		VCD	1
**High (n = 11)**	Early; NK1RA + 5-HT_3_RA + DEX, Delayed; NK1RA + 5-HT_3_RA + DEX	CDDP + CPT-11	4
		FEC	2
		EC	2
		R-CHOP	3

Tmab; Trastuzumab, VNR; vinorelbine, MTX; Methotrexate, VCR; Vincristine, PSL; Prednisolone, GEM; Gemcitabine, nab-PTX; nab-Paclitaxel, DTX; Docetaxel, EMP; estramustine, Bmab; Bevacizumab, sLV5FU2; simplified biweekly 5-FU & leucovorin, VTD; bortezomib, thalidomide, dexamethasone, CBDCA; Carboplatin, FOLFOX6; combination of Oxaliplatin and 5FU, CapeOX; capecitabine plus intermittent oxaliplatin, SOX; S-1 plus intermittent oxaliplatin, FOLFIRI; combination of Irinotecan and 5FU IRIS; CPT-11; Irinotecan, VCD; bortezomib, Cyclophosphamide, dexamethasone, CDDP; Cisplatin, FEC; 5FU, Epirubicin, Cyclophosphamide, EC; Epirubicin, Cyclophosphamide, R-CHOP; Rituximab, Cyclophosphamide, Doxorubicin, Vincristine, Prednisolone.

### Compliance with the antiemetic guideline

The compliance of the antiemetic guideline was examined. Our anti-emetic guideline is indicated in Table 3. Sufficient antiemetic treatments were performed for the minimal-risk regimens in all patients. In the low-risk group, all except 1 case received sufficient treatments; one under treatment was performed in the early phase, and no under treatments were performed in the delayed phase. For the moderate-risk group, sufficient treatments were performed in 14 of cases (82.4%); no under treatments occurred in the early phase, whereas three under treatments were performed in the delayed phase. For the high-risk group, sufficient treatments were performed in 6 cases (54.5%); no under treatments occurred in the early phase, whereas five under treatments were performed in the delayed phase. The overall compliance rate was 87.7%; the compliance rate was 98.6% with early phase CINV. The compliance rate for delayed phase CINV was 87.7%; specifically, 97.4% in the low-risk group, 82.4% in the moderate group, and 54.5% in the high-risk group.

**Table 3:** **Compliance of antiemetic treatment**

**Emetogenisity**	**Actual measure (Early)**	**Actual measure (Delayed)**	**n**	**Compliance**
**Minimal**	5-HT_3_RA + DEX	no	2	Sufficient
	5-HT_3_RA	no	1	Sufficient
	DEX	no	1	Sufficient
	no	no	3	Sufficient
**Low**	5-HT_3_RA + DEX	no	29	Sufficient
	5-HT_3_RA + DEX	DEX	3	Sufficient
	5-HT_3_RA + DEX	D2RA	1	Sufficient
	DEX	DEX	1	Sufficient
	DEX	no	3	Sufficient
	no	no	1	**Insufficient**
**Moderate**	5-HT_3_RA + DEX	DEX	6	Sufficient
	5-HT_3_RA + DEX	NK1RA + DEX	3	Sufficient
	NK1RA + 5-HT_3_RA + DEX	NK1RA	2	Sufficient
	NK1RA + 5-HT_3_RA + steroid (MM)	NK1RA	1	Sufficient
	NK1RA + 5-HT_3_RA + DEX	5-HT_3_RA + DEX + NK1RA	1	Sufficient
	5-HT_3_RA + DEX	no	3	**Insufficient**
	5-HT_3_RA + DEX	DEX + D2RA	1	Sufficient
**High**	NK1RA + 5-HT_3_RA + DEX	NK1RA	2	**Insufficient**
	NK1RA + 5-HT_3_RA + steroid (ML)	NK1RA	2	Sufficient
	NK1RA + 5-HT_3_RA + steroid (ML)	NK1RA + D2RA	1	Sufficient
	5-HT_3_RA + DEX	DEX	2	**Insufficient**
	5-HT_3_RA + DEX	no	1	**Insufficient**

5-HT_3_RA; 5-HT_3_ receptor antagonist, DEX; Dexamethasone, D2RA; Dopamine receptor D2 receptor antagonist, NK1RA; Neurokinin 1 receptor antagonist, MM; multiple myeloma, ML; malignant lymphoma.

### Comparison between patient outcome and compliance with the guidelines

**Figure 2:**
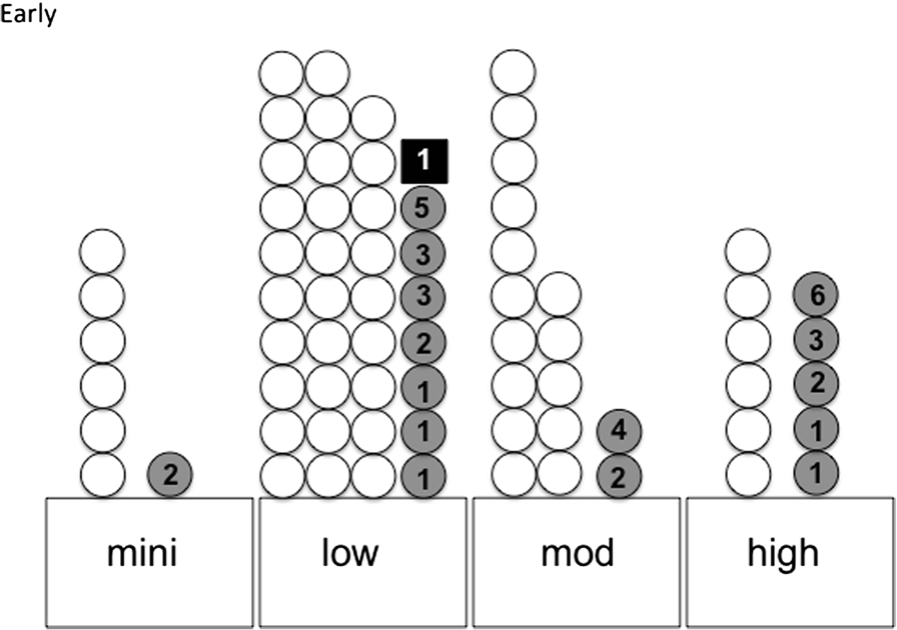
**Patient-reported outcome regarding early CINV in patients treated with sufficient antiemetic guidelines.** Mini, minimal-risk group; mod, moderate-risk group. An open circle indicates the case with a complete response. The shaded circle indicates CIN (the number corresponds to the grade of nausea). The closed rectangle indicates CIV (the number corresponds to the number of times of vomiting).

We next compared the patient-reported CINV and the compliance of guideline. As shown in Figure 2, early CINV was not prevented in 22.2% of cases, although the sufficient antiemetic treatment against was conducted. However, early CINV was not prevented at all in the under treatment case. The overall relative risk for compliance with the guidelines was calculated to be 0.22 (*P* < 0.05). Specifically, in the minimal-risk group, CIN was not prevented in one of 7 cases (14.3%), in spite of sufficient treatment (the grade of nausea was 2 (G2)). In the low-risk group, CIN was not prevented in 8 of 37 cases (21.6%) (G1 to G5; median = G2.5). Among these, one patient vomited once (2.7%). In the moderate-risk group, CIN was not prevented in 2 of 7 cases (11.8%) (G2 to G4; median = G3), but no CIV was observed. In the high-risk group, CIN was not prevented in 5 of 11 cases (45.5%) (G1 to G6; median = G3), but no CIV was observed.

**Figure 3:**
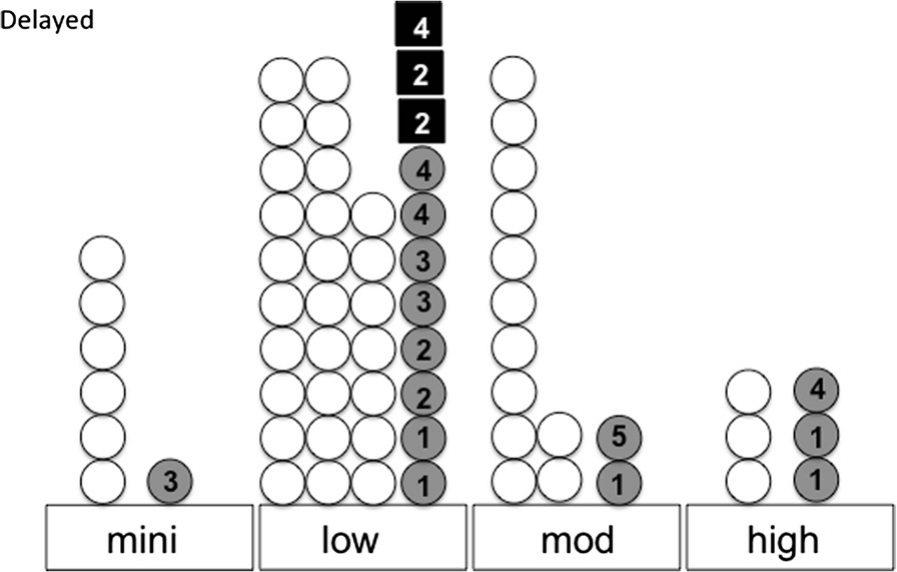
**Patient-reported outcome regarding delayed CINV in patients treated with sufficient antiemetic guidelines.** Mini, minimal-risk group; mod, moderate-risk group. An open circle indicates the case with a complete response. The shaded circle indicates CIN (the number corresponds to the grade of nausea). The closed rectangle indicates CIV (the number corresponds to the number of times of vomiting).

Delayed CINV was not prevented in 17 of the 65 cases (26.2%), even though sufficient antiemetic treatments were conducted (Figure 3). In the under treatment cases, CINV (G1) was not prevented in 1 of 8 cases (12.5%). The overall relative risk for delayed CINV was 2.22 (*P* < 0.05). Specifically, in the minimal-risk group, CIN was not prevented in 1 of 7 cases (14.3%) (G3). In the low-risk group, CIN was not prevented in 11 of 38 cases (29.0%) (G1 to G4; median = G3). Among these, three patients vomited (two patients twice and one patient once). In the moderate-risk group, CIN was not prevented in 2 of 14 cases (14.3%) (G1 to G5; median = G3), but there were no instances of vomiting. In the high-risk group, CIN was not prevented in 3 of 6 cases (50%) (G1 to G4; median = G4), but there was no vomiting.

In this observational study, CIV occurred only in the patients that received low emetogenic chemotherapeutic agents. One male patient (71 years old) that reported early CIV was treated for the second time with nab-pactlitaxel for his carcinoma of unknown origin, mediastinal lymph node metastasis, and pleural dissemination. The remaining three patients claimed breakthrough CIV. Of these, one female patient (42 years old) was treated for the fourth time with eribulin for breast cancer with bone and liver metastases. Another female patient (61 years old) was treated with docetaxel for the second time for lung cancer with asymptomatic and multiple micrometastases in the brain. Finally, one male patient (55 years old) was treated for the twelfth time with docetaxel for lung cancer and cancerous pleuritis. The compliance rate of our hospital was better than that reported previously [10]. Preventive treatment against early CINV was almost completely effective; however, the prevention of delayed CINV was not sufficient in the high-risk group according to the current guidelines. It is necessary to prevent delayed CINV. According to the patient-reported outcome, delayed CINV occurred in only 12.5% of the patients that were treated with anti-emetic agents insufficiently. However, early and delayed CIN occurred in 22.2% and 26.2% of patients, respectively, in spite of conduction of sufficient anti-emetic treatment, particularly, in the high-risk group, early and delayed CIN occurred in 45.5% and 50.0% of patients, respectively. However, the grade of CIN was not so severe (The median grade of early CIN = G3. and delayed CIN = G4). Moreover, no CIV (breakthrough CIV) was reported. The guidelines for the prevention of CINV in the high-risk group appeared to be effective to comparable extent. In the low-risk group, early and delayed CINV occurred in 21.6% and 28.9% of patients, respectively, in spite of sufficient anti-emetic treatment (The median grade of the early and the delayed CIN = G2.5 and G3, respectively). Moreover, 3 cases of breakthrough CIV were reported. Among these, all three vomited several times. These data suggest that compliance with the guideline alone could not prevent emetogenicity. Consistent with this, a previous study also reported that sufficient measures could not prevent breakthrough CIV completely, even in low-risk patients [11]. In addition, some reports have discussed the difficulty of prevention using antiemetic guidelines for delayed CINV [12,13]. In particular, prevention in low-risk patients remains controversial.

Current guidelines are based primarily on the emetogenicity of chemotherapeutic agents. Data suggested that individual differences in the sensitivity to antiemetic treatments may occur. There are also too many individual parameters, including exposure to chemotherapy, alcohol use, age, and gender [14,15]. As such, data should be gathered and analyzed regarding CINV cases. Such factors may include individual sensitivities to the preventive agents, tumor status, and the physical condition of patient. Refining the antiemetic measures is also necessary; accordingly, some methods have been proposed [13,16]. However, the establishment of the personalized precautions as well as the standard one is likely to be necessary for the complete prevention of CINV.

## Conclusions

The generation of antiemetic guidelines might contribute toward patient compliance with antiemetic measures. However, complete prevention remains challenging because individual factors should be considered.

## Additional file

Additional file 1: Table S1. Detailed characteristics of participants.

## Abbreviations

QOL: Quality of life
CINV: Chemotherapy-induced nausea and vomiting
5-HT3: 5-hydroxytryptamine
5-HT3RA: 5-HT3 receptor antagonists
NK1: Neurokinin 1
NK1RA: NK1 receptor antagonist
MASCC: Multinational association of supportive care in cancer
ASCO: the American society of clinical oncology
NCCN: National comprehensive cancer network
DEX: Dexamethasone
MAT: MASCC antiemesis tool
