# Supplementary material for: Cohort study of consistency between the compliance with guidelines for chemotherapy-induced nausea and vomiting and patient outcome
Source: BMC Pharmacol Toxicol. 2015 Mar 28;16:5. doi: 10.1186/s40360-015-0005-1 (PMC4379596; doi:10.1186/s40360-015-0005-1)
Supplement: Additional file 1: Table S1. — Detailed characteristics of participants. [file 40360_2015_5_MOESM1_ESM.doc]

**Additional file 1: Table S1.** Detailed characteristics of participants

| gender | diagnosis | stage | previous surgery | previous chemotherapy | conducted regimen | emetogenicity |
| --- | --- | --- | --- | --- | --- | --- |
| female | br | IV | no | EC, DTX, Capecitabine, NVB | GEM | low |
| male | crc | IV | no | no | Bmab+CapeOX | moderate |
| female | br | IA | yes | no | FEC | high |
| female | br | IV | no | no | Tmab+PTX | low |
| male | crc | IV | yes | FOLFIRI | sLV5FU2 | low |
| male | crc | IV | yes | S-1, SOX, Bmab+IRIS | CPT-11 | moderate |
| female | cup | IV | no | CBDCA+DTX, CBDCA+PTX,  CBDCA+GEM | GEM | low |
| male | rcc | IV | yes | sunitinib | temsirolimus | minimal |
| male | crc | IIIB | yes | FOLFOX6 | sLV5FU2 | low |
| male | crc | IV | yes | Bmab+FOLFOX6 | Bmab+sLV5FU2 | low |
| male | lu | IV | no | CBDCA+PTX, PEM | DTX | low |
| female | lu | IV | no | CBDCA+PTX, crizotinib, PEM | DTX | low |
| female | ga | IV | no | S-1+DTX | CPT-11+CDDP | high |
| male | ga | IV | yes | SP, CPT-11 | PTX | low |
| male | lu | IIIB | no | CBDCA+PTX | DTX | low |
| female | panc | IV | no | GEM | GEM+nab-PTX | low |
| female | crc | IV | no | FOLFIRI, FOLFOX6 | sLV5FU2 | low |
| male | ga | IA | yes | S-1 | nab-PTX | low |
| female | ga | IV | no | SP, S-1+DTX | CPT-11 | moderate |
| female | br | IIB | yes | FEC | Tmab+PTX | low |
| female | crc | IV | yes | Cmab+FOLFOX6 | Bmab+FOLFIRI | moderate |
| male | pro | IV | no | LHRH agonist+antiandrogen | DTX+PSL | low |
| male | eso | III | yes | FP | DTX | low |
| male | pro | IV | no | LHRH agonist+antiandrogen | DTX+PSL | low |
| male | crc | II | yes | CapeOX | SOX | moderate |
| female | br | IIB | yes | no | EC | high |
| female | br | IIIC | yes | FEC, Tmab+DTX | Tmab+VNR | minimal |
| male | crc | IV | yes | FOLFIRI, sLV5FU2, FOLFOX6 | Bmab+IRIS | moderate |
| female | ml | I | no | no | R-CHOP | high |
| male | ga | II | no | no | S-1+DTX | low |
| male | mm | I | no | Rd | VCD | moderate |
| male | lu | IV | no | CBDCA+PTX, gefitinib, erlotinib, PEM | DTX | low |
| male | ga | IIIA | yes | S-1, Tmab+XP, nab-PTX, CPT-11 | DTX | low |
| female | cup | IV | yes | GEM | DTX | low |
| male | lu | IV | no | no | Bmab+CBDCA+PTX | moderate |
| female | br | IV | no | Tmab+PTX | Tmab | minimal |
| male | pro | I | yes | LHRH agonist | DTX+PSL | low |
| male | ga | IIIA | yes | S-1+DTX | CPT-11+CDDP | high |
| male | cup | IV | no | CBDCA+PTX | nab-PTX | low |
| male | pro | II | yes | LHRH agonist+antiandrogen | DTX+EMP | low |
| female | panc | III | no | S-1+GEM | GEM | low |
| female | br | IV | no | PTX, FEC, TC, Tmab+NVB | eribulin | low |
| male | eso | II | yes | CDGP+5FU | DTX | low |
| female | br | IIIB | yes | FEC, DTX, Tmab+GEM | Tmab+VNR | minimal |
| female | panc | IV | no | no | GEM | low |
| male | panc | III | yes | no | GEM | low |
| female | crc | IIB | yes | FOLFIRI | IRIS | moderate |
| female | panc | III | no | no | GEM | low |
| female | br | IIB | yes | PTX, NVB, GEM, DTX, eribulin | nab-PTX | low |
| female | all | - | no | CPM+DNR+VCR+L-ASP, Ara-C+ETP+DEX, MTX+6MP, | MTX+VCR+PSL | minimal |
| male | ccc | IVA | yes | no | GEM | low |
| female | br | IIA | yes | TC | Tmab | minimal |
| male | ga | III | yes | S-1 | CDDP+CPT-11 | high |
| female | cup | IV | no | SP | CDDP+CPT-11 | high |
| male | panc | IV | no | no | GEM | low |
| male | mm | I | no | no | VTD | low |
| female | lu | IIIA | yes | CBDCA+DTX, PEM | VNR | minimal |
| female | crc | IIIA | yes | no | CapeOX | moderate |
| female | crc | IIIA | yes | no | CapeOX | moderate |
| male | pro | IV | no | LHRH agonist+antiandrogen | DTX+PSL | low |
| male | ml | I | no | no | R-CHOP | high |
| female | ga | IIIB | yes | S-1 | S-1+DTX | low |
| male | lu | IIA | yes | UFT/uzel, gefitinib, CBDCA+PEM, PEM | Bmab+CBDCA+PTX | moderate |
| female | br | IIA | yes | no | EC | high |
| female | br | IIA | yes | no | FEC | high |
| female | crc | IIIB | yes | FOLFOX6 | IRIS | moderate |
| male | crc | IIIA | yes | no | CapeOX | moderate |
| female | lu | IV | no | gefitinib | Bmab+CBDCA+PTX | moderate |
| female | crc | IV | no | CapeOX | Pmab+FOLFOX6 | moderate |
| male | crc | IV | yes | IRIS | Pmab+FOLFOX6 | moderate |
| male | ml | IV | no | no | R-CHOP | high |
| female | lu | IV | no | gefitinib, Bmab+CBDCA+PTX, PEM | DTX | low |
| female | panc | III | no | no | S-1+GEM | low |
|  |  |  |  |  |  |  |

br; breast cancer, crc; colorectal cancer, cup; cancer unknown primary, rcc; renal cell carcinoma, lu; lung cancer,

ga; gastric cancer, panc; pancreas cancer, pro; prostate cancer, eso; esophageal cancer, ml; malignant lymphoma,

all; acute lymphoid leukemia, ccc; cholangiocellular carcinoma,
